# A multivariable risk prediction model for pregnancies of uncertain viability without a fetal pole: a prospective cohort study

**DOI:** 10.3389/fmed.2026.1814757

**Published:** 2026-05-08

**Authors:** Chee Wai Ku, Marie Min Tse Tan, Yu Bin Tan, Ting Yu Chang, Celeste Hong Fei Lim, Hiu Gwan Chan, Thiam Chye Tan, Jerry Kok Yen Chan, See Ling Loy

**Affiliations:** 1 Department of Reproductive Medicine, KK Women’s and Children’s Hospital, Singapore, Singapore; 2 Duke-NUS Medical School, Singapore, Singapore; 3 Maternal and Child Health Research Institute, KK Women’s and Children’s Hospital, Singapore, Singapore; 4 Yong Loo Lin School of Medicine, National University of Singapore, Singapore, Singapore; 5 Paediatric Endocrinology Service, KK Women’s and Children’s Hospital, Singapore, Singapore; 6 Obstetrics and Gynaecology Academic Clinical Programme (OBGYN ACP), SingHealth Duke-NUS Academic Medical Centre, Singapore, Singapore; 7 Department of Obstetrics and Gynaecology, KK Women’s and Children’s Hospital, Singapore, Singapore

**Keywords:** clinical triage tool, early pregnancy loss prediction, pregnancies of uncertain viability, risk prediction model, serum progesterone

## Abstract

**Introduction:**

Pregnancy of uncertain viability affects up to 30% of early pregnancies. Among women presenting with per-vaginal bleeding and absence of a fetal pole, prognosis remains indeterminate. The lack of validated prediction tools contributes to clinical uncertainty and maternal anxiety.

**Methods:**

This prospective cohort study was conducted at KK Women’s and Children’s Hospital, Singapore, from October 2017 to May 2023. We recruited 446 women with single intrauterine pregnancies between 5 and 12 weeks’ gestation by last menstrual period (LMP), presenting with either per-vaginal bleeding or abdominal pain and pregnancy of uncertain viability without a fetal pole. Predictor variables included maternal age, ethnicity, nausea, history of miscarriage, serum progesterone, and gestational age by LMP. The primary outcome was spontaneous miscarriage by 16 weeks. Multivariable logistic regression models were evaluated using the area under the receiver operating characteristic curve (AUROC) and Akaike information criterion. Risk scores derived from β-coefficients were categorized into low (≤1), medium (1–4), and high (4–7) risk groups.

**Results:**

Among 1,938 women screened, 446 were eligible, and 187 (41.9%) had early pregnancy loss. Low serum progesterone (<35 nmol/L; OR 22.0, 95% CI 13.2–36.5), absence of nausea (2.34, 1.34–4.08), greater gestational age (2.26, 1.87–2.72), and older maternal age (1.08, 1.04–1.12) independently predicted early pregnancy loss. The four-factor model achieved an AUROC of 0.88; the corresponding risk-score model achieved 0.86, with sensitivity 0.74 and specificity 0.88. Early pregnancy loss rates were 11.5%, 33.8%, and 88.2% across risk groups.

**Discussion:**

A simple four-factor model accurately predicts early pregnancy loss in women with pregnancy of uncertain viability without a fetal pole, supporting early risk stratification and individualized counseling.

## 1 Introduction

Pregnancy of uncertain viability is a common diagnostic challenge in early obstetric care, often presenting with vaginal bleeding (1). Affecting up to 30% of early pregnancies, pregnancy of uncertain viability is defined by transvaginal ultrasound (TVUS) findings of an intrauterine gestational sac without a visible fetal pole, or the presence of a fetal pole without detectable fetal cardiac activity (2, 3). The availability of highly sensitive home pregnancy tests has led to earlier pregnancy detection, prompting women to seek formal ultrasound assessments before definitive sonographic signs are present (4). Especially for women with per-vaginal bleeding, the diagnosis of pregnancy of uncertain viability increases anxiety and for the clinician, there is significant prognostic ambiguity. Current management involves serial ultrasounds spaced 7–14 days apart (5–9). Despite variations in clinical guidelines, all necessitate a waiting period and reassessment before confirming a diagnosis. This interim phase of diagnostic uncertainty can cause profound physical and psychological distress for women and their families (2, 4, 10–13).

Many studies have explored various markers to predict early pregnancy loss. Clinical variables such as maternal age, gestational age and pictorial bleeding assessment chart (PBAC) scores, radiological parameters such as mean sac diameter (MSD), yolk stalk sign and presence of fetal heart, and serum biomarkers such as beta human chorionic gonadotropic (hCG) levels, progesterone and estradiol have shown potential (2, 4, 14–18). However, existing models often focus on isolated variables (15, 16, 18), a few sets of blood tests with serial measurements (14, 19) or a combination of clinical history and ultrasound scan, often requiring detailed imaging or extensive data collection which may not be practical in routine care (2, 4, 17, 20). To date, none has integrated clinical history and blood tests into a simple, unified prognostic tool. This gap leaves clinicians without effective interim guidance in early pregnancy. A model that captures the interplay of key risk factors could improve prognostication and enable timely, personalized care.

This study aims to develop a novel multivariable risk-score model to predict early pregnancy loss among women presenting with per vaginal bleeding or abdominal pain with pregnancy of uncertain viability without a fetal pole. This represents an earlier developmental stage, where prognosis of the pregnancy is uncertain without established sonographic criteria to guide further management. The model integrates clinical and biochemical factors. We also evaluate the performance of various models that incorporate different sets of these predictors to allow flexible adaptation across diverse healthcare contexts, including those with limited resources.

## 2 Materials and methods

### 2.1 Study participants and procedures

This single-center cohort study utilized prospective data from women presenting with either per-vaginal bleeding or abdominal pain, and pregnancy of uncertain viability without a fetal pole at the Urgent O&G Centre (UOGC) of KK Women’s and Children’s Hospital (KKH) between 6*^th^* October 2017 and 20*^th^* May 2023, in Singapore. We included women with a single confirmed intrauterine pregnancy between 5- and 12-weeks’ gestation dated via last menstrual period (LMP) attending our UOGC for per-vaginal bleeding or abdominal pain, with a diagnosis of pregnancy of uncertain viability with an intrauterine gestational sac without a fetal pole on TVUS. Exclusion criteria included women who received progestogens during the current pregnancy, those with multiple gestations, *in vitro* fertilization (IVF) pregnancy, incomplete, missed, or inevitable miscarriage, pregnancy of unknown location, ectopic pregnancy, termination of pregnancy and those with a fetal pole in the current pregnancy. At the initial visit, the attending clinician conducted a targeted history taking, physical examination, and ultrasound evaluation. Serum progesterone was measured as routine clinical practice. History focused on questions related to the presenting complaint. In line with the clinical management protocol in UOGC, women with serum progesterone below 35 nmol/L were considered “high-risk” for early pregnancy loss (21) and were treated with oral dydrogesterone according to the manufacturer’s protocol (Duphaston, Abbott), provided anticipatory guidance, and monitored closely. Conversely, women with serum progesterone levels of 35 nmol/L or higher were classified as “low-risk” and managed conservatively with counseling and reassurance, without the use of progestogens. All study participants were reviewed in the outpatient clinic within 2 weeks and followed up until 16 weeks’ gestation to ascertain pregnancy outcome, via review of medical records.

### 2.2 Assessment of exposure variables

Maternal age, ethnicity (Chinese, Malay, Indian, Others), number of previous miscarriages, presence of pain and nausea were recorded at the time of recruitment via an investigator-administered questionnaire in either English or Chinese. Maternal body mass index (BMI) was calculated using each patient’s measured height (in cm) and weight (in kg) on the day of their visit. BMI was categorized as underweight, normal weight, overweight and obese (<18.5, 18.5–24.9, 25–29.9, ≥30 kg/m^2^) according to the World Health Organization BMI criteria (22). Per-vaginal bleeding was quantified using a pictorial blood assessment chart, which rates the extent of staining on disposable sanitary products on a scale from 0 to 4 (23). Maternal blood samples were collected at the recruitment visit in plain tubes and centrifuged for 10 min at 3,000 *g* within 2 h of collection, and serum progesterone was subsequently measured in the KKH clinical laboratory using a commercial ARCHITECT progesterone kit (Abbott, Ireland). Gestational age was determined by the first day of their last menstrual period.

### 2.3 Assessment of pregnancy outcome

The primary outcome was early pregnancy loss, defined as self-reported uterine evacuation following an inevitable or incomplete miscarriage, or complete miscarriage with an empty uterus by 16 weeks’ gestation (21). Most miscarriages occur before week 16 of gestation, and other etiologies of late miscarriage, such as cervical insufficiency, are unlikely to be predicted in early pregnancy (24). Data were retrieved from medical records.

### 2.4 Assessment of sample size

To develop a multivariable prediction model, maternal age, progesterone levels, number of miscarriages, gestational age by LMP (weeks) are treated as continuous variables. Absence of nausea and ethnicity have two categories each. According to Machin and Campbell (25), 10 × 8 = 80 early pregnancy loss events was required. There were 187 participants with early pregnancy loss in the study, which was sufficient for the development of the prediction model.

### 2.5 Statistical analyses

Baseline maternal demographics and pregnancy characteristics were compared between women with ongoing pregnancies and those who had an early pregnancy loss by 16 weeks’ gestation. Continuous variables were analyzed using two-sample *t*-tests and presented as means with standard deviations. Categorical variables were analyzed using Chi-square tests and presented as frequencies and percentages.

Missing data were addressed using multiple imputation by chained equations with fully conditional specification (26). Univariable and multivariable logistic regression models with Firth correction were used to identify predictors of early pregnancy loss, with results expressed as odds ratios (ORs) and 95% confidence intervals (CIs). Model performance was evaluated using the area under the receiver operating characteristic (AUROC) curve, based on different combinations of pre-selected maternal characteristics. The characteristics were selected based on literature review, clinical judgment, a direct acyclic graph, and corroboration with univariable analyses (*p* < 0.15) (27). These included (a) clinical characteristics such as maternal age (28), ethnicity, absence of nausea (29), number of previous miscarriages, gestational age by LMP; and (b) serum progesterone levels (21, 30, 31). The AUROC value of >0.80 indicates that the model exhibits a good discriminatory ability (32). The lowest Akaike information criterion (AIC) indicates the best fit based on existing data.

A full model (Model 1) was developed with all candidate predictors. Non-significant predictors were removed to generate a reduced model (Model 2). To assess the incremental value of biochemical data, serum progesterone was excluded from Models 1 and 2 to create Models 3 and 4, which included only clinical predictors. The final model was chosen to develop a risk score model based on the criteria of having the highest AUROC and the lowest AIC.

For the risk score, age was categorized into below 35 and 35 years or older (33). The optimal cut off point of gestational age in receiver operating curve (ROC) analysis was determined with Youden index. A score was assigned to each variable based on the range of β coefficients estimated from the multivariable logistic model. A score of 0 was assigned to the reference category of each variable. The risk scores was then categorized into tertiles and named as lower risk (≤1), medium risk (>1 to 4) and higher risk (>4 to 7), according to the prevalence of early pregnancy loss in respective groups. The model’s cut-off point was determined when specificity, positive predictive value (PPV) and negative predictive value (NPV) of the model exceeded 80%.

A 4-fold cross-validation was conducted to estimate predictive ability and performance generalizability. The 95% CIs of AUROC were calculated using bootstrapping with 2,000 replications. Sensitivity analysis was performed on complete dataset (*n* = 365). Statistical significance was set at *p*-value < 0.05 and all tests were two-tailed. Statistical analyses were performed using Stata 18 (Stata Corporation) and R packages “mice” and “pROC.”

### 2.6 Ethics approval

This study was reviewed and approved by the SingHealth Centralized Institutional Review Board (CIRB) of Singapore (CIRB Reference no. 2017/2638). The Transparent Reporting of a multivariable prediction model for Individual Prognosis or Diagnosis (TRIPOD) guideline was followed and written informed consent was obtained from all participants enrolled in the study (34). All research described in this manuscript adhered to the Declaration of Helsinki (35).

## 3 Results

### 3.1 Maternal characteristics

Among 1,938 women with per-vaginal bleeding or abdominal pain screened, the number of patients presenting with pregnancy of uncertain viability at 16 weeks’ gestation was 28.1% (544/1,938) (Supplementary Figure 1). We excluded women with a fetal pole (*n* = 1,353), those who received progestogen treatment during the current pregnancy (*n* = 2), had multiple gestations (*n* = 6), pregnancy of unknown location (*n* = 3), ectopic pregnancy (*n* = 2), *in vitro* fertilization pregnancy (*n* = 2), or had undergone termination of pregnancy (*n* = 26). Of the 544 women enrolled, 98 women were lost to follow-up. Of the included women eligible for analyses, 41.9% (187/446) experienced early pregnancy loss by 16 weeks, while 58.1% (259/446) had an ongoing pregnancy.

Comparison of baseline maternal characteristics by early pregnancy loss status showed that women who had early pregnancy loss were older (32.5 vs. 30.8 years), more likely to be of non-Chinese ethnicity (59.4% vs. 44.0%), and had a greater gestation age by LMP (7.5 vs. 6.2 weeks). They were also more likely to report absence of nausea (87.7% vs. 72.0%), to have low serum progesterone levels (72.7% vs. 10.8%) (Table 1).

**TABLE 1:** Baseline maternal demographics by early pregnancy loss status at 16 weeks’ gestation for women with pregnancies of uncertain viability without a fetal pole with per-vaginal bleeding or abdominal pain.

Characteristic	All (*N* = 446)	Pregnancy status at 16 weeks’ gestation		*P*-value
		Ongoing pregnancy (*N* = 259)	Early pregnancy loss (*N* = 187)	
Clinical factors				
Maternal age, years (SD)	31.6 (4.77)	30.8 (4.17)	32.5 (5.36)	<0.001
Ethnicity, *n* (%)				<0.001
Chinese	221 (49.6)	145 (56.0)	76 (40.6)	–
Malay	111 (24.9)	60 (23.2)	51 (27.3)	–
Indian	58 (13.0)	30 (11.6)	28 (15.0)	–
Others	56 (12.6)	24 (9.3)	32 (17.1)	–
Maternal BMI, kg/m^2^, *n* (%)	25.0 (5.57)	25.0 (5.44)	25.0 (5.79)	0.751
Underweight (<18.5)	20 (5.4)	12 (5.3)	8 (5.6)	0.967
Normal (18.5–24.9)	193 (52.2)	116 (51.3)	77 (53.5)	–
Overweight (25.0–29.9)	95 (25.7)	60 (26.5)	35 (24.3)	–
Obese (≥30.0)	62 (16.8)	38 (16.8)	24 (16.7)	–
History of miscarriage, *n* (%)				0.690
0	358 (80.6)	208 (80.3)	150 (81.1)	–
1	70 (15.8)	43 (16.6)	27 (14.6)	–
≥2	16 (3.6)	8 (3.1)	8 (4.3)	–
Gestation age by LMP, weeks (SD)	6.7 (1.43)	6.2 (1.05)	7.5 (1.54)	<0.001
Nausea, *n* (%)				<0.001
No	290 (78.6)	154 (72.0)	136 (87.7)	–
Yes	79 (21.4)	60 (28.0)	19 (12.3)	–
Pain, *n* (%)				0.286
No	254 (58.5)	142 (54.8)	112 (59.9)	–
Yes	180 (41.5)	117 (45.2)	75 (40.1)	–
PBAC score, *n* (%)				0.312
0	7 (1.6)	5 (2.0)	2 (1.1)	–
1	426 (95.9)	248 (96.5)	178 (95.2)	–
2	11 (2.5)	4 (1.6)	7 (3.7)	–
Biochemical factors				
Serum progesterone, *n* (%)				<0.001
<35 nmol/L	164 (36.8)	28 (10.8)	136 (72.7)	–
≥35 nmol/L	282 (63.2)	231 (89.2)	51 (27.3)	–

Continuous variables were analyzed via a two-sample *t*-test and presented as mean values with standard deviations, while categorical variables were analyzed using Chi-square test and are presented as frequencies and percentages. BMI, body mass index; LMP, last menstrual period; PBAC, pictorial bleeding assessment chart; UOGC, Urgent Obstetrics and Gynecology Centre. Missing data: maternal BMI (*n* = 76); Gestational age by last menstrual period (*n* = 3); history of miscarriage (*n* = 2); nausea (*n* = 77); PBAC score (*n* = 2); progesterone treatment given at the UOGC (*n* = 1).

### 3.2 Univariable analysis of risk factors and multivariable prediction models for early pregnancy loss

Univariable analyses showed that among clinical factors, absence of nausea had the highest odds ratio (OR 2.34; 95% CI: 1.34–4.08). Overall, low serum progesterone was the strongest individual predictor (OR 22.0; 95% CI: 13.2–36.5) of early pregnancy loss (Supplementary Table 1).

Table 2 shows multivariable prediction models for early pregnancy loss at 16 weeks which incorporated various combinations of clinical and biochemical factors. Model 1, which included all six factors, yielded an AUROC of 0.88 (95% CI: 0.84–0.91). Two non-significant predictors were removed to produce Model 2, which retained four factors (maternal age, absence of nausea, gestational age by LMP, and serum progesterone). It achieved a similar AUROC of 0.88 (95% CI: 0.84–0.91). Model 3, which included only clinical factors, yielded an AUROC of 0.81 (95% CI: 0.76–0.85). For Model 4, two non-significant clinical factors were removed, resulting in the lowest performance with an AUROC of 0.80 (95% CI: 0.76–0.84). For parsimony, Model 2 was chosen as the final model for risk-score development. Sensitivity analysis using the complete dataset showed similar results (Supplementary Table 2).

**TABLE 2:** Multivariable prediction models for early pregnancy loss at 16 weeks in patients with pregnancy of uncertain viability with absent fetal pole with imputed data (*n* = 446).

Characteristic	Model 1 (*n* = 446)	Model 2 (*n* = 446)	Model 3 (*n* = 446)	Model 4 (*n* = 446)
	aOR (95% CI)			
Clinical factors				
Maternal age (years)	1.09 (1.03–1.16)	1.09 (1.03–1.15)	1.08 (1.03–1.14)	1.07 (1.02–1.13)
Non-Chinese	1.45 (0.86–2.45)	–	1.62 (1.03–2.54)	–
Absence of nausea	2.54 (1.20–5.40)	2.43 (1.15–5.12)	2.91 (1.52–5.59)	2.82 (1.48–5.38)
Number of miscarriages	1.00 (0.67–1.51)	–	0.96 (0.68–1.35)	–
Gestational age by LMP (weeks)	1.57 (1.26–1.94)	1.59 (1.29–1.97)	2.26 (1.85–2.76)	2.31 (1.9–2.82)
Biochemical factors				
Low serum progesterone levels (<35 nmol/L)	13.9 (7.92–24.5)	14.2 (8.11–25.0)	–	–
AUROC (95% CI)	0.88 (0.84–0.91)	0.88 (0.84–0.91)	0.81 (0.76–0.85)	0.80 (0.76–0.84)
AIC	292.7	290.4	363.8	363.3

Multivariable analyses of clinical and blood test variables for early pregnancy loss risk at 16 weeks in a cohort of patients with pregnancy of uncertain viability with absent fetal pole. Characteristics analyzed include maternal age, ethnicity, absence of nausea, number of miscarriages, gestational age by LMP and serum progesterone levels. Adjusted odds ratios (aOR) and 95% confidence intervals (CI) are presented. The aOR is adjusted odds ratio for all other factors in the given model. The area under the receiver operating characteristic curve (AUROC) values and Akaike information criterion (AIC) for each model were presented. The 95% CI of AUROC was calculated using bootstrapping with 2,000 replications.

### 3.3 Risk-score model for early pregnancy loss

The risk-score model had a total score range between 0 and 7 (Table 3). We determined the optimal cut-off point for gestational age as 6.5 weeks (sensitivity = 0.69, specificity = 0.75, PPV = 66.7%, NPV = 77.3%). Individual risk scores were calculated using the following formula: Early pregnancy loss risk score = 2 (age ≥ 35 years) + 3 (serum progesterone level < 35 nmol/L) + 2 (gestation age by LMP ≥ 6.50 weeks) + 1 (absence of nausea).

**TABLE 3:** Risk score model for early pregnancy loss at 16 weeks’ gestation (*n* = 446).

Characteristic	aOR (95% CI)	β coefficient	Score
Maternal age (years)			
<35	1.00	–	0
≥35	2.35 (1.8–14.48)	0.85	1
Nausea			
No	2.49 (1.13–5.46)	0.91	1
Yes	1.00	–	0
Gestational age by LMP (weeks)			
<6.50	1.00	1.07	0
≥6.50	2.92 (1.70–5.00)	–	2
Serum progesterone levels			
<35 nmol/L	15.36 (8.75–26.96)	2.73	3
≥35 nmol/L	1.00	–	0

Coefficient of logistic regression model, adjusted odds ratios (aOR) and 95% confidence intervals (CI) are presented. The risk score values were estimated based on the range of β coefficients in the model. Score 1: β < 1.00; Score 2: β was between 1.00 and 2.00; Score 3: β > 2.00. The model at optimal cut off point had sensitivity of 0.74, specificity of 0.88. Average AUC calculated from cross validation: 0.86. The 95% CI of AUROC (0.83–0.90) was calculated using bootstrapping with 2,000 replications.

**FIGURE 1:**
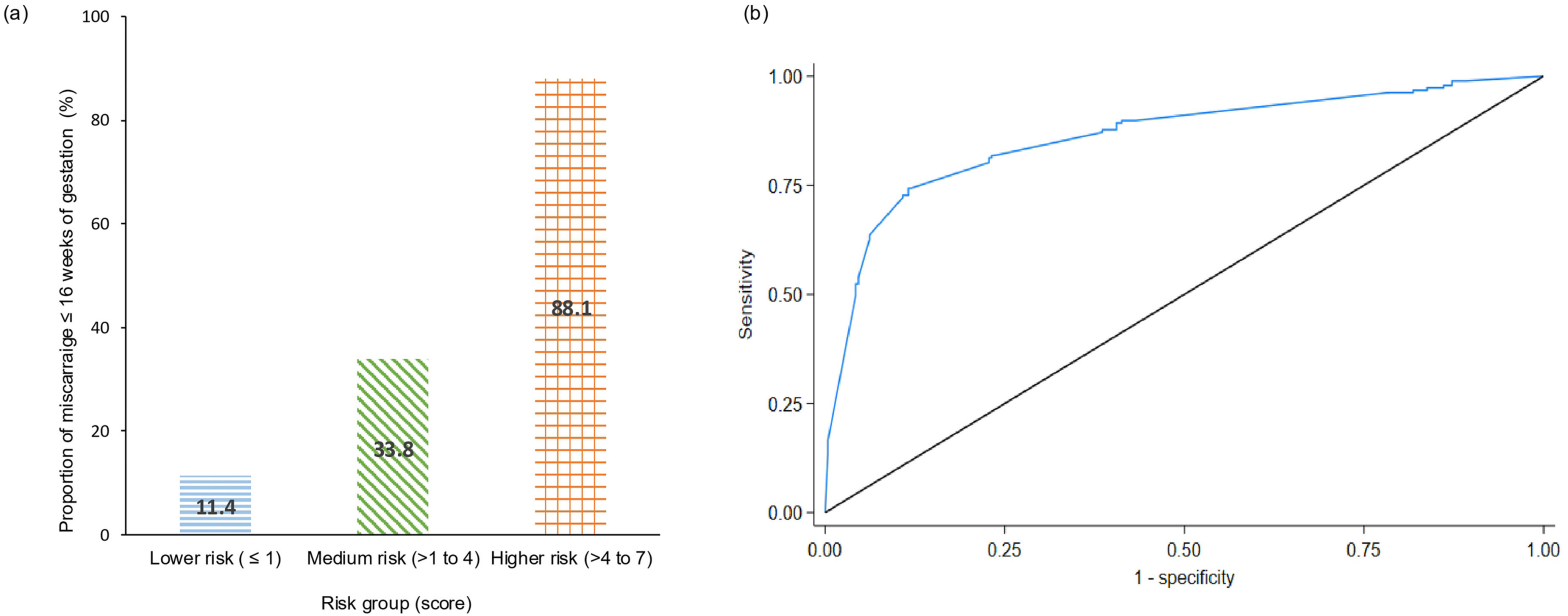
**(a)** Proportion of early pregnancy loss by 16 weeks’ gestation stratified by low- (≤ 1), medium- (1–4) and high- risk (5–7) group **(b)** ROC performance risk score model.

The proportion of women who experienced early pregnancy loss was 11.5% in the low-risk group (score ≤ 1), 33.8% in the medium risk group (score > 1–4), and 88.2% in the high-risk group (score > 4–7) (Figure 1). The risk-score model demonstrated good discriminatory ability, achieving an AUROC of 0.86. The model had sensitivity and specificity values of 0.74 and 0.88, respectively, at the cut-off point of 4. Detailed performance metrics including sensitivity, specificity, PPV and NPV are presented in Supplementary Table 3.

## 4 Discussion

### 4.1 Main findings

This study developed and internally validated risk prediction models for early pregnancy loss among women with pregnancy of uncertain viability without a fetal pole, a clinically important subgroup characterized by high prognostic uncertainty. By using four routinely available factors, including maternal age, nausea, gestational age by LMP, and serum progesterone, we constructed a clinically relevant and easy-to-use risk-score model that enables early risk stratification without a clear ultrasound prognosis. While several individual risk factors for early pregnancy loss are well-established, our findings demonstrate their combined predictive value, highlighting the importance of integrating multiple factors to improve prediction accuracy.

### 4.2 Interpretation

Our findings underscore the importance of incorporating serum progesterone into risk assessment for early pregnancy viability. Serum progesterone emerged as the strongest individual predictor, outperforming all clinical variables when used in isolation. Its biological role in maintaining the endometrium, promoting uterine quiescence, and supporting early placental development underpins its predictive value (36). While prior studies have examined serum progesterone in predicting early pregnancy loss in the first trimester, and among those with threatened miscarriage and ectopic pregnancy, few have incorporated it into practical and validated risk prediction tools (19, 37–40).

Among clinical factors, the absence of nausea was the most significant predictors in our cohort, consistent with existing evidence suggesting that nausea is a reassuring symptom of pregnancy viability (29, 41–46). Nausea likely reflects elevated hCG levels produced by the placenta, and progesterone may further exacerbate nausea by reducing smooth muscle tone and delay gastric emptying (47). Advanced maternal age was a significant demographic risk factor and its association with increased incidence of embryonic aneuploidy is well-established (45, 48–52). Although accuracy of gestational age by LMP may be limited by recall bias or irregular cycles, it remained a significant predictor (53), especially in pregnancy of uncertain viability cases where no fetal pole is visible and LMP is often the only available dating method. A diagnosis of pregnancy of uncertain viability without a fetal pole at a more advanced gestational age by LMP suggests delayed embryonic development that could increase the risk of early pregnancy loss (54, 55).

Interestingly, neither the history of miscarriages nor body mass index were significant predictors of early pregnancy loss and did not enhance our model performance. While prior studies in preconception and early pregnancy cohorts (56–60) have demonstrated being overweight or obese as a risk factor for early pregnancy loss, it was not observed in our population. Similarly, although a history of miscarriage is a known risk factor – particularly in women with three or more prior losses which may suggest an underlying genetic or physiological cause (52, 61–63), there was no significant association found in our study. This is possibly attributable to the relatively small proportion (<20%) of women who reported a prior miscarriage and an even smaller subgroup with two or more miscarriages (<3.6%).

Several studies have created predictive tools for pregnancy of uncertain viability, often relying on singular variables like ultrasound features or blood tests, which may lack sufficient discriminatory power in isolation (4, 14–16, 18, 19, 64). One model had multiple clinical and ultrasound variables (20). Elson et al.’s model combined maternal age, mean sac diameter (MSD), and serum progesterone in a cohort of 200 women, achieving an AUC of 0.97 and an externally validated AUC of 0.85 (65, 66). Bottomley et al. later proposed a formal risk scoring system that excluded serum progesterone and incorporated PBAC, yolk sac and gestational age by LMP (2, 67). However, reliance on MSD limits generalizability and feasibility in low-resource settings as it is not routinely measured in many clinical settings, including ours, due to its dependence on high resolution ultrasound and operator expertise. Furthermore, model performance declined when gestational age was excluded, further emphasizing the limitations of ultrasound-dependent models (17, 68). In contrast, our models showed strong predictive power with the inclusion of serum progesterone, even in the absence of ultrasound data, reinforcing its potential as a key biomarker in pregnancy of uncertain viability assessment. Moreover, our models include symptoms like nausea and LMP-based gestational age, which are easier to obtain and reproduce across clinical environments.

Our findings have several clinical implications. The proposed risk-score model is designed to be applicable across diverse healthcare populations, including those with limited access to ultrasound imaging. The model incorporates both clinical and biochemical variables, which enables early triage of women with pregnancy of uncertain viability without the need to wait for serial ultrasound reassessments. Routine serum progesterone testing should be considered as part of this approach, given its strong predictive value. The model supports prompt risk stratification, facilitating early counseling, expectation management, and timely intervention to reduce psychological distress during diagnostic uncertainty. Additionally, the availability of multiple model iterations, with and without biochemical inputs, offers flexibility for clinicians to select the most appropriate tool based on local resources and clinical context, including settings with limited access to imaging or laboratory testing. These models provide timely, evidence-based frameworks to support personalized care for women with pregnancy of uncertain viability. Future research should consider incorporating psychosocial and lifestyle variables, and externally validate our risk-score model across different populations to confirm generalizability and refine model calibration. Future studies could externally validate Model 4 in low-resource settings to enhance generalizability.

### 4.3 Strength and limitations

This study is one of the first prospective evaluation of a large multi-ethnic cohort of women with pregnancy of uncertain viability without a fetal pole, with cross-validation to enhance predictive ability and performance generalizability. To our knowledge, no previous studies of this scale have comprehensively examined clinical and biochemical risk factors for early pregnancy loss among women with pregnancy of uncertain viability without a fetal pole, nor evaluated the interplay of these factors within risk-prediction models capable of stratifying early pregnancy loss risk before 16 weeks of gestation. The multi-ethnic composition of our study population enhances its generalizability to Asian settings. The availability of multiple model iterations strengthens the model’s adaptability across healthcare systems with varying diagnostic capacity, including resource-limited settings.

However, our study is limited by the lack of data on potential confounders such as smoking, alcohol use, and other modifiable lifestyle factors. While we used LMP to estimate gestational age, we did not collect data on menstrual regularity or recall reliability, which may influence dating accuracy. We also did not assess psychological outcomes and patient-centered experiences, which are relevant in the context of early pregnancy loss risk. Additionally, we did not include MSD and presence of yolk sac in our models. As this study focused on pregnancy of uncertain viability without a fetal pole, a clinically important group where prognostic uncertainty is greatest, we included pregnancies regardless of yolk sac status to reflect the spectrum of early presentations. This enhances generalizability of our models. Although these variables have been used in previous prediction tools, their reliance on high-resolution ultrasound and operator expertise limits feasibility and generalizability, particularly in low-resource settings. While internal cross-validation was performed to assess the robustness of our findings, future studies with an independent cohort should be conducted for external validation.

### 4.4 Conclusion

This study presents a clinically relevant and easily applicable risk-score model, derived from four key clinical and biochemical predictors, to predict early pregnancy loss risk in women with pregnancy of uncertain viability without a fetal pole. Developed from the best-performing multivariable model, the risk score enables early risk stratification and personalized counseling. Additionally, alternative models excluding biochemical variables were evaluated, supporting flexible implementation across diverse clinical and resource settings. These tools have the potential to improve early triage, guide clinical decision-making, and enhance outcomes for targeted women presenting with pregnancy of uncertain viability.

## Data Availability

The raw data supporting the conclusions of this article will be made available by the authors, without undue reservation.
