# Supplementary material for: A multivariable risk prediction model for pregnancies of uncertain viability without a fetal pole: a prospective cohort study
Source: Front Med (Lausanne). 2026 May 8;13:1814757. doi: 10.3389/fmed.2026.1814757 (PMC13194374; doi:10.3389/fmed.2026.1814757)
Supplement: Supplementary file 1 [file Table_1.docx]

**Supplemental Table 1.** Univariable analysis of risk factors for early pregnancy loss at 16 weeks in patients with pregnancy of uncertain viability with absent fetal pole (n=446)

| Factor Type | Factor | Univariable analyses (95% CI) | AUROC (95% CI) |
| --- | --- | --- | --- |
| Clinical | Maternal age (years) | 1.08 (1.04-1.12) | 0.59 (0.54-0.65) |
|  | Non-Chinese | 1.86 (1.27-2.72) | 0.58 (0.53-0.62) |
|  | Absence of Nausea | 2.34 (1.34-4.08) | 0.58 (0.54-0.63) |
|  | Number of miscarriage | 1.02 (0.76-1.37) | 0.50 (0.46-0.54) |
|  | Gestational Age by LMP (weeks) | 2.25 (1.87-2.72) | 0.78 (0.73-0.82) |
| Biochemical | Low serum progesterone levels (<35nmol/L) | 22.0 (13.2-36.5) | 0.81 (0.77-0.85) |

**Supplemental Table 2.** Multivariable Prediction models for early pregnancy loss at 16 weeks in patients with pregnancy of uncertain viability with absent fetal pole with complete dataset (n= 365)

| Characteristic | Model 1  (n = 365) | Model 2  (n = 365) | Model 3  (n = 365) | Model 4  (n = 365) |
| --- | --- | --- | --- | --- |
|  | aOR  (95% CI) | | | |
| Clinical Factors | | | | |
| Maternal age (years) | 1.09 (1.03-1.16) | 1.09 (1.02-1.16) | 1.09 (1.03-1.15) | 1.09 (1.03-1.14) |
| Non-Chinese | 1.19 (0.66-2.15) |  | 1.34 (0.81-2.19) |  |
| Absence of Nausea | 3.34 (1.53-7.31) | 3.28 (1.51-7.13) | 2.91 (1.52-5.59) | 3.52 (1.8-6.88) |
| Number of miscarriages | 1.02 (0.62-1.67) |  | 1.00 (0.67-1.5) |  |
| Gestational Age by LMP (weeks) | 1.53 (1.21-1.93) | 1.54 (1.23-1.94) | 2.19 (1.77-2.70) | 2.22 (1.8-2.74) |
| Biochemical Factors | | | | |
| Low serum progesterone levels (<35nmol/L) | 15.4 (8.28-28.7) | 15.6 (8.36-28.91) |  |  |
| AUROC (95% CI) | 0.88 (0.85-0.92) | 0.88 (0.85-0.92) | 0.81 (0.76-0.85) | 0.81 (0.76-0.85) |
| AIC | 316.1 | 310.7 | 401.9 | 397.6 |

**Supplemental Table 3.** Sensitivity, specificity, PPV and NPV of Multivariable predictive models for early pregnancy loss at 16 weeks in patients with threatened miscarriage at different threshold) (n=446)

| Threshold of risk score | Sensitivity | Specificity | PPV | NPV |
| --- | --- | --- | --- | --- |
| ≥1 | 98.6% | 13.8% | 45.2% | 93.1% |
| ≥2 | 89.3% | 57.9% | 60.5% | 88.2% |
| ≥3 | 81.5% | 76.7% | 71.7% | 85.2% |
| ≥4 | 74.2% | 88.4% | 82.2% | 82.6% |
| ≥5 | 63.4% | 93.8% | 88.1% | 78.0% |
| ≥6 | 53.6% | 95.5% | 89.6% | 74.0% |
| ≥7 | 16.0% | 99.6% | 96.8% | 62.2% |

Abbreviations: PPV, Positive Predicted Value; NPV, Negative Predictive Value.

**
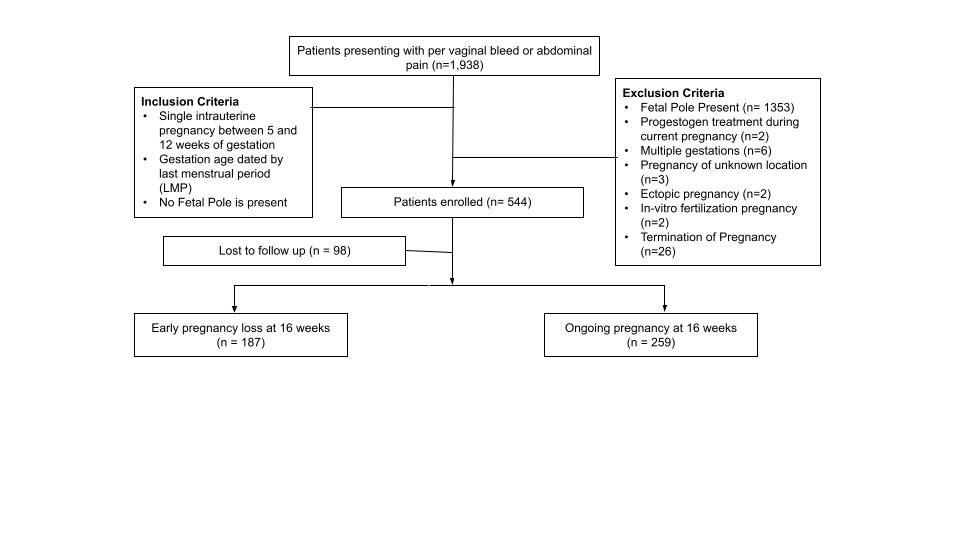
**

**Supplemental Figure 1.** Flowchart of clinical outcomes of patients with pregnancy of uncertain viability with absent fetal pole.

**Supplemental Table 1.** Univariable analysis of risk factors for early pregnancy loss at 16

weeks in patients with threatened miscarriage (n=446)

**Supplemental Table 2.** Multivariable predictive models for early pregnancy loss at 16 weeks in patients with threatened miscarriage using the complete dataset (n=365)

**Supplemental Table 3.** Multivariable predictive models for early pregnancy loss at 16 weeks in patients with threatened miscarriage using the complete dataset (n=446)

**Supplemental Figure 1.** Flowchart of clinical outcomes of patients with pregnancy of uncertain viability with absent fetal pole.
